# Redox Chronobiology of Secondary Hyperparathyroidism in Chronic Kidney Disease: Exploring the Circadian–Melatonin–PTH Axis

**DOI:** 10.3390/antiox15101235

**Published:** 2026-09-24

**Authors:** Hsuan-Chu Hsu, Chia-Chao Wu, Kuo-Cheng Lu

**Affiliations:** 1 Division of Nephrology, Department of Medicine, Yangming Branch, Taipei City Hospital, Taipei 111024, Taiwan or grana7349@gmail.com; 2 Faculty of Medicine, School of Medicine, National Yang Ming Chiao Tung University, No. 155, Section 2, Linong St, Taipei 11221, Taiwan; 3 Division of Nephrology, Department of Internal Medicine, Tri-Service General Hospital, National Defense Medical University, Taipei 114202, Taiwan or chiachaowu@gmail.com; 4 Department and Graduate Institute of Microbiology and Immunology, National Defense Medical University, Taipei 114201, Taiwan; 5 Division of Nephrology, Department of Medicine, Taipei Tzu Chi Hospital, Buddhist Tzu Chi Medical Foundation, New Taipei City 23142, Taiwan; 6 Division of Nephrology, Department of Medicine, Fu-Jen Catholic University Hospital, New Taipei City 24352, Taiwan

**Keywords:** chronic kidney disease, circadian rhythm, immune dysfunction, inflammation, melatonin, mitochondrial ROS, NADPH oxidase, NRF2–KEAP1, oxidative stress, RANKL, secondary hyperparathyroidism, vascular calcification

## Abstract

The novelty of this review lies in its falsifiable, time-resolved framework that integrates the parathyroid molecular clock with melatonin loss, compartment-specific redox failure, and bidirectional PTH–immune signalling rather than treating these mechanisms as parallel associations. Secondary hyperparathyroidism (SHPT) in chronic kidney disease (CKD) is conventionally attributed to phosphate retention, calcitriol deficiency, hypocalcaemia, and fibroblast growth factor 23 (FGF23) resistance. We synthesise evidence linking mitochondrial reactive oxygen species (ROS), NADPH oxidase (NOX) activation and impaired nuclear factor erythroid 2-related factor 2 (NRF2)–Kelch-like ECH-associated protein 1 (KEAP1) signalling to uraemic toxins, inflammation, hyperphosphataemia and dialysis-related oxidant stress. Sustained ROS engage NF-κB, MAPK and endoplasmic reticulum stress pathways that drive parathyroid dysfunction, RANKL-dependent osteoclastogenesis, endothelial dysfunction and vascular smooth-muscle-cell calcification. Inflammation is treated as bidirectional: immune activation amplifies ROS and mineral-tissue injury, while sustained PTH may reciprocally modify monocyte/macrophage, neutrophil and lymphocyte function—an immunological arm that remains hypothesis-generating given sparse, uraemia-confounded evidence in CKD–SHPT. We convert this synthesis into four testable predictions: CKD-stage-specific uncoupling of 24-h PTH and melatonin profiles; redox-dependent loss of chief-cell mineral sensing; cell-specific immune effects of PTH at patient-relevant concentrations; and differential responses to timed versus conventional SHPT therapy. Melatonin may oppose redox and inflammatory injury through receptor-dependent and mitochondrial actions. Because physiological ROS are essential signalling mediators, we emphasise redox balance rather than maximal antioxidant capacity, distinguish human, animal and cellular evidence, and flag supraphysiological PTH, phosphate and melatonin exposures. The proposed axis is a mechanistic research framework, not a basis for routine melatonin, antioxidant or immune-directed therapy pending dedicated CKD outcome trials.

## 1. Introduction

As kidney function declines, phosphate excretion and calcitriol synthesis become impaired, FGF23 rises early, αKlotho falls, and hypocalcaemia and sustained parathyroid hormone (PTH) elevation emerge with advancing disease [1,2,3,4]. These changes define the mineral-metabolic component of CKD-mineral and bone disorder (CKD–MBD) and promote secondary hyperparathyroidism (SHPT), which is characterised by increased PTH secretion, parathyroid hyperplasia and, in advanced disease, partial autonomy [3,5,6].

The consequences of SHPT extend beyond mineral biochemistry. Sustained PTH exposure promotes high-turnover bone disease through osteoblast- and osteocyte-derived RANKL, while PTH1R signalling also has recognised actions in cardiovascular cells [5,7,8]. These effects occur within a uraemic environment marked by hyperphosphataemia, retained toxins, inflammation, mitochondrial dysfunction and dialysis-related oxidant generation, providing a plausible setting in which redox stress can amplify skeletal and cardiovascular injury [9,10,11,12]. Inflammation is predominantly an upstream driver, although experimental and ex-vivo data suggest that sustained PTH may also modify monocyte/macrophage, neutrophil and lymphocyte responses; the direction and independence of these effects remain uncertain because of confounding by uraemia, phosphate, vitamin D status and dialysis exposure [6,13,14]. Accordingly, this review focuses on mitochondrial and NADPH oxidase-derived ROS, nuclear factor erythroid 2-related factor 2 (NRF2)–Kelch-like ECH-associated protein 1 (KEAP1) defence, and downstream redox-sensitive signalling rather than treating oxidative stress as a single undifferentiated process [5,15].

Chronobiology adds a temporal dimension to this biology. PTH secretion and bone turnover are rhythmic, and experimental work demonstrates an intrinsic molecular clock in the parathyroid gland; advanced CKD is also associated with disruption of the nocturnal melatonin signal [16,17,18,19,20,21,22]. Melatonin is therefore relevant not only as a chronobiotic but also as a mitochondrial and redox regulator with receptor-dependent actions in bone and vascular tissues [11,12,16,17,23,24,25].

We integrate these observations into a single working model: CKD-associated circadian disruption may weaken nocturnal and mitochondrial protection, while mineral stress, inflammation and uraemic oxidants increase redox burden in parathyroid, skeletal, immune and cardiovascular compartments. Sustained PTH and tissue injury may then reinforce the same inflammatory and oxidative environment. The evidence supporting individual links is unequal, particularly for reciprocal PTH–immune signalling and for direct coupling between melatonin loss and parathyroid clock dysfunction. We therefore distinguish human observations from experimental inference and interpret therapeutic hypotheses in light of the neutral unadjusted primary cardiovascular result of EVOLVE [14,25,26,27].

## 2. Circadian Organisation of PTH Secretion and Melatonin Signalling

### 2.1. Core Clock Architecture

The suprachiasmatic nucleus (SCN) acts as the central circadian pacemaker and synchronises cell-autonomous peripheral clocks through neural, endocrine and behavioural signals (Figure 1) [28]. At the molecular level, BMAL1 (ARNTL) and CLOCK activate *Period* (PER1/2) and *Cryptochrome* (CRY1/2) transcription; accumulated PER and CRY proteins subsequently inhibit BMAL1/CLOCK activity, generating an approximately 24-h transcriptional cycle [29]. A second interlocking loop, involving REV-ERBα/β (NR1D1/2) and RORα, stabilises the oscillator and couples circadian timing to metabolism [30]. Clock machinery is also present in peripheral tissues relevant to CKD–MBD, including bone and kidney; the parathyroid clock is considered separately below [31,32].

### 2.2. Endogenous PTH Rhythm and the Parathyroid Clock

In healthy adults, intact PTH exhibits a reproducible diurnal pattern, rising during the biological night, peaking in the early morning and falling to a late-morning nadir [16,18]. PTH secretion is also pulsatile, with a substantial proportion released in low-amplitude bursts at intervals of approximately 10–20 min [19]. The circadian component persists under controlled conditions, supporting an endogenous rhythm rather than a simple response to meals or calcium intake [18]. Experimental studies further demonstrate rhythmic clock-gene expression in parathyroid tissue and disruption of this clock in CKD–MBD models [17]. Whether altered PTH timing contributes independently to skeletal disease in human CKD remains uncertain and requires direct 24-h profiling across CKD stages [16,20].

### 2.3. Melatonin as a Chronobiotic and Redox-Regulatory Hormone

Melatonin is secreted predominantly during the biological night and conveys circadian information to peripheral tissues [21]. Its high-affinity receptors, MT1 (MTNR1A) and MT2 (MTNR1B), mediate tissue-specific actions; vascular responses differ between receptor subtypes, while MT2 has been implicated in osteoblast differentiation and inhibition of osteoclastogenesis [23,33,34]. Melatonin also has receptor-independent and receptor-dependent antioxidant and anti-inflammatory effects and can influence mitochondrial redox homeostasis [21,22]. In bone models, melatonin intersects with clock machinery and RANKL/OPG regulation, including REV-ERBα-dependent suppression of osteoclastogenesis [16,24,35].

## 3. The Parathyroid Chief Cell: Synthesis, Secretion and Hyperplasia

Parathyroid chief cells integrate extracellular calcium, vitamin D and FGF23 signals through CaSR, VDR and the FGFR1–Klotho complex. In CKD, progressive loss of these regulatory pathways accompanies parathyroid hyperplasia and reduced responsiveness to inhibitory signals (Figure 2) [36]. This section focuses on those features that are most relevant to the proposed circadian–redox framework while retaining the established mineral biology of SHPT [6].

### 3.1. Molecular Control and FGF23 Signalling

Extracellular ionised calcium provides the dominant minute-to-minute signal through CaSR, a class-C G-protein-coupled receptor that engages Gαq/phospholipase C and Gαi pathways; unlike most secretory systems, CaSR activation suppresses PTH release [3,4]. Over longer intervals, calcitriol–VDR signalling suppresses *PTH* transcription, phosphate promotes *PTH* synthesis and secretion, and FGF23 acts through FGFR1-associated pathways that include canonical MAPK signalling and a calcineurin-dependent branch [36,37]. In parathyroid-specific Klotho knockout mice, residual FGF23 responsiveness is calcineurin-dependent, indicating that FGF23 signalling is not exclusively Klotho-dependent; the relevance of this pathway to calcineurin-inhibitor exposure after transplantation remains mechanistically plausible rather than clinically established [6,37,38].

### 3.2. Hyperplasia and Receptor Downregulation in CKD

Persistent secretory stimulation is accompanied by parathyroid growth, initially as diffuse hyperplasia and, in advanced SHPT, as nodular proliferation [36,39]. Hyperplastic and especially nodular tissue shows reduced expression of CaSR, VDR, FGFR1, and Klotho, together with increased proliferative activity [36,39,40]. These changes have functional consequences: hyperplastic tissue becomes less responsive to FGF23 and other inhibitory inputs [36,39,41]. Reduced CaSR shifts the calcium–PTH response, VDR loss weakens transcriptional suppression, and disruption of Klotho–FGFR1 signalling further impairs FGF23 feedback; experimental combined loss of Klotho and CaSR accelerates parathyroid hyperplasia [39]. Thus, advanced SHPT reflects both sustained stimulation and progressive loss of regulatory restraint, helping to explain the reduced responsiveness of nodular disease to medical therapy [6,36,39].

### 3.3. A Peripheral Clock and a Candidate Redox Target

Parathyroid tissue also contains an intrinsic circadian oscillator. Experimental CKD–MBD disrupts clock-gene rhythmicity in the gland, suggesting that uraemia may alter both the amount and timing of PTH secretion [17]. By contrast, direct evidence that oxidative stress independently drives receptor loss or hyperplasia in human parathyroid chief cells is sparse; redox biology should therefore be treated as a candidate mechanism rather than an established cause [36,39,40]. These circadian and redox questions should be studied alongside, rather than substituted for, established SHPT therapies and outcome data [25,26,32,36].

## 4. PTH Signalling in Bone and Its Redox Modulation

PTH effects on bone are comparatively well characterised. PTH acts directly on osteoblast-lineage cells, with osteocytes representing an important in-vivo target, whereas osteoclast activation is indirect. The biological response depends strongly on the duration and pattern of PTH1R stimulation, and oxidative stress can modify both bone-forming and bone-resorbing pathways [15].

### 4.1. PTH1R Signalling and the Anabolic–Catabolic Switch

PTH and PTH-related peptide signal through the class-B receptor PTH1R, activating Gs-adenylyl cyclase-PKA–CREB and Gαq-phospholipase-C–PKC pathways in target cells [8,9]. Temporal exposure is critical: intermittent PTH favours osteoblast activity and net bone formation, whereas sustained exposure shifts remodelling toward osteoclastogenesis and bone loss [8,42,43]. This difference partly reflects signalling kinetics, because anabolic early-response genes are transient, whereas maximal induction of resorptive mediators such as RANKL requires more prolonged stimulation [44]. SHPT therefore resembles the sustained rather than intermittent pattern, although skeletal PTH responsiveness is modified by CKD stage and bone-turnover state [15].

### 4.2. Osteocytes, RANKL/OPG and FGF23

Osteocytes are central mediators of PTH-dependent remodelling: osteocytic PTH1R signalling contributes to both anabolic and resorptive responses [15,45]. PTH suppresses osteocytic sclerostin, thereby relieving inhibition of Wnt/β-catenin signalling [8,46], and osteocytes are a major source of the RANKL that drives PTH-dependent osteoclast formation in vivo [45,47,48]. Mature osteoclasts do not require direct PTH1R stimulation; instead, PTH acts through osteoblast- and osteocyte-derived RANKL, with OPG providing a competing decoy signal [8,47,48]. In CKD, this pathway operates within a more complex skeletal environment that includes PTH resistance, FGF23 excess, uraemic toxins and treatment-related low-turnover states; accordingly, KDIGO assessment of renal osteodystrophy is not based on PTH concentration alone [3,15,49].

### 4.3. Melatonin as a Counter-Regulatory Signal in Bone

Melatonin can oppose several pathways associated with PTH-driven bone resorption in experimental systems. Predominantly through MT2-linked signalling, it promotes osteoblast-lineage differentiation and survival and suppresses osteoclastogenesis, including effects on the OPG/RANKL balance, NF–κB and REV–ERBα pathways [23,24,33]. Human evidence is limited and derives mainly from non-CKD populations, where small trials have reported changes in bone-turnover activity and modest improvements in bone mineral density [33,50]. These findings support biological plausibility but do not establish fracture prevention or efficacy in renal osteodystrophy. Many experimental studies also use melatonin concentrations well above physiological nocturnal levels, which limits direct translation to CKD [51].

## 5. CKD–SHPT as an Integrated Circadian–Melatonin–Redox Disorder

### 5.1. Mineral, Circadian and Melatonin Inputs

The parathyroid response to CKD begins as an adaptive attempt to maintain mineral balance but becomes progressively maladaptive. Phosphate retention, reduced calcitriol synthesis and rising FGF23 interact to increase *PTH* secretion [1,2,3], while chronic stimulation promotes gland hyperplasia and loss of CaSR, FGFR1 and Klotho, reducing sensitivity to normal feedback [36,39]. These mineral changes become more pronounced as CKD advances [3,17]. In parallel, advanced CKD and dialysis are frequently associated with attenuation, phase disturbance or loss of the normal nocturnal melatonin signal [21,52,53,54]. Reported melatonin patterns are not completely uniform, however, because altered production, sleep–light disruption and impaired clearance may produce different concentration profiles across studies [53,54]. Sleep-wake disruption and abnormal nocturnal blood-pressure patterns are common in dialysis populations [52,53,54]. Whether melatonin dysrhythmia is mechanistically coupled to disruption of the parathyroid clock in patients remains untested and should be regarded as a central hypothesis of this review rather than an established clinical mechanism [6,17]. The integrated components of the proposed axis are summarised in Table 1.

### 5.2. Central Hypothesis and Feed-Forward Architecture

The proposed model links circadian disturbance, melatonin dysregulation, oxidative stress, immune activation and SHPT without assuming that each component has equal evidential support. Circadian disruption and reduced nocturnal melatonin could alter mitochondrial function and antioxidant responses; uraemic toxins, phosphate and inflammation concurrently increase mitochondrial and NOX-derived ROS. These signals may converge on redox-sensitive inflammatory, osteogenic and apoptotic pathways in parathyroid, immune, skeletal and cardiovascular cells. A reciprocal effect of sustained PTH on immune-cell function remains plausible but unproven [6,13]. The model therefore predicts that biological effects depend not only on mean PTH and phosphate concentrations but also on timing, cell type and local redox state [11,12,17,22]. Figure 3 summarises this architecture and distinguishes established observations from hypothesis-generating links [14,25,27].

## 6. Oxidative Stress in the Circadian–Melatonin–PTH Axis

### 6.1. Mitochondrial ROS and NADPH Oxidase Activation

Mitochondria and NADPH oxidases are major interacting sources of ROS in CKD. Electron-transport-chain dysfunction can increase superoxide and hydrogen peroxide generation, while NOX activity can amplify mitochondrial oxidant production and redox-sensitive signalling. NOX1, NOX2 and NOX4 have been particularly implicated in renal and vascular injury. Importantly, ROS are not uniformly harmful: low, spatially restricted concentrations participate in receptor signalling, autophagy, antimicrobial defence and adaptive stress responses. Oxidative pathology arises when production exceeds the buffering and repair capacity of the relevant cellular compartment (Figure 4) [12,27,55,56,61].

### 6.2. Uraemic Toxins, Inflammation, Phosphate and Dialysis

Protein-bound uraemic toxins, particularly indoxyl sulfate and p-cresyl sulfate, promote oxidative and inflammatory injury and may suppress NRF2-regulated defence [12,57]. Hyperphosphataemia is another important redox stimulus: experimental high-phosphate models show increased mitochondrial ROS, NF-κB activation and osteogenic signalling in vascular smooth-muscle cells, although commonly used in-vitro phosphate exposures may exceed usual circulating concentrations [58,59]. Haemodialysis can lower retained solutes but also produces episodic oxidative stress through blood–membrane interactions, leukocyte and complement activation, iron exposure and changes in oxygenation; the magnitude of this effect varies with treatment context [12]. These processes are established upstream contributors to inflammation and tissue injury, whereas direct reciprocal effects of PTH on immune cells remain less certain [14,62].

### 6.3. Impaired NRF2–KEAP1 Antioxidant Signalling

Under physiological stress, oxidation of KEAP1 cysteines stabilises NRF2, allowing transcription of haem oxygenase-1, NAD(P)H quinone dehydrogenase 1, glutathione-synthesis enzymes, thioredoxin and other cytoprotective programmes. Experimental chronic renal failure shows impaired NRF2 activation despite increased oxidative pressure, creating a paradoxical state of high ROS with inadequate inducible defence [61]. Uraemic toxins and sustained inflammation further disturb NRF2–KEAP1 signalling and favour NF-κB dominance [12,57]. In the proposed axis, loss of nocturnal melatonin may compound this defect, because melatonin supports mitochondrial redox homeostasis and can activate endogenous antioxidant enzymes rather than acting only as a direct scavenger [22,27,56]. Mechanistically, NRF2 is the master transcriptional regulator of the cellular antioxidant response. Under basal conditions, KEAP1 binds NRF2 and targets it for cullin-3-dependent ubiquitination and proteasomal degradation; oxidation or electrophilic modification of reactive KEAP1 cysteines releases NRF2, which translocates to the nucleus, dimerises with small MAF proteins and binds antioxidant-response elements (AREs) to transactivate a coordinated cytoprotective programme—haem oxygenase-1, NAD(P)H quinone dehydrogenase 1, the glutamate–cysteine-ligase subunits (GCLC/GCLM) and other glutathione-synthesis enzymes, thioredoxin and the cystine transporter xCT (SLC7A11) [27,61]. This pathway is reciprocally coupled to inflammation: NRF2 restrains NF-κB-driven cytokine transcription, whereas NF-κB activation suppresses ARE signalling. Thus, blunted NRF2 output in CKD simultaneously lowers antioxidant capacity and de-represses inflammatory signalling, reinforcing the high-ROS/low-defence state central to this axis [12,27]. In chronic renal failure, NRF2 activation is impaired despite mounting oxidative pressure, in part because protein-bound uraemic toxins such as indoxyl sulfate and p-cresyl sulfate downregulate NRF2 and its target genes; NRF2 additionally exerts context- and tissue-dependent actions that may be protective or, when persistently or excessively activated, maladaptive, cautioning against indiscriminate pharmacological NRF2 induction [56,57]. Restoration of physiological, compartment-appropriate NRF2 signalling—rather than blanket antioxidant loading—is therefore the rational redox target, and provides a mechanism through which loss of the nocturnal melatonin signal, a physiological NRF2 activator, could further weaken adaptive defence [22,27].

### 6.4. Redox Biology of the Parathyroid Chief Cell

Direct evidence for abnormal redox signalling in human uraemic parathyroid chief cells is limited. The gland is nevertheless exposed to the same phosphate, inflammatory mediators and oxidised solutes that affect other CKD tissues. A testable model is that sustained ROS may perturb mitochondrial function, calcium handling and proteostasis and thereby facilitate loss of mineral sensing or proliferative selection; at present, however, these mechanisms have not been shown to cause parathyroid hyperplasia independently in patients [12,36,39,56]. Table 2 summarises the cell-specific oxidative and immune hypotheses and indicates where evidence is indirect.

### 6.5. Bidirectional PTH–Inflammation–Immunity Crosstalk

Inflammation is clearly an upstream source of oxidative stress in CKD, but sustained PTH may also modify immune-cell function. PTH-responsive and calcium-sensitive pathways have been described in several immune-cell populations, and experimental or ex-vivo studies report changes in intracellular calcium, proliferation, cytokine production, phagocytosis and oxidative burst. These findings are heterogeneous, however, and vary with species, cell type, PTH fragment, concentration and exposure duration; many studies were performed in uraemia or primary hyperparathyroidism, making direct PTH effects difficult to isolate [6,13,14].

In monocytes and macrophages, PTH-related calcium signalling could intersect with NF-κB, inflammasome and ROS pathways, but uraemic toxins are themselves strong activators of macrophage inflammation and therefore major confounders [14]. Neutrophil calcium handling may influence chemotaxis, phagocytosis and oxidative burst, whereas lymphocyte studies have reported changes in activation, proliferation and antibody responses. The direction and reproducibility of these effects are inconsistent, and concentration-resolved data from patients with CKD–SHPT remain sparse [6,13,14].

Elevated PTH should therefore be considered a possible modifier of immune dysfunction rather than an established independent cause. Phosphate retention, uraemic toxins, dialysis-related complement activation, vitamin Ddeficiency, comorbidity and oxidative stress can each alter innate and adaptive immunity. Demonstrating a specific PTH effect will require cell-specific studies at patient-relevant exposures and interventional evidence that selective PTH lowering reverses immune abnormalities independently of broader improvement in the uraemic milieu [6,13,14].

### 6.6. Osteoblasts, Osteocytes and the RANKL Axis

Excess ROS can impair osteoblast differentiation and matrix production, disrupt mitochondrial function and shift stress-responsive FOXO signalling away from Wnt/β-catenin pathways. Osteocytes are likewise vulnerable to mitochondrial and oxidative injury, and osteocyte apoptosis can promote local remodelling. ROS and inflammatory cytokines also enhance NF-κB and MAPK signalling, increasing RANKL-dependent osteoclastogenesis [44,47,48,51]. In uncontrolled SHPT, sustained PTH therefore provides a persistent resorptive stimulus while oxidative stress may simultaneously weaken bone-forming cells, although the magnitude of this interaction in human CKD remains uncertain [51,62].

### 6.7. Endothelium, Cardiomyocytes and VSMCs

Uraemic oxidants can impair endothelial nitric-oxide bioavailability, promote inflammatory adhesion programmes and increase cell injury. In cardiomyocytes, mitochondrial dysfunction and abnormal calcium handling may converge with PTH1R- and FGF23-dependent hypertrophic signalling. In vascular smooth-muscle cells, phosphate, mitochondrial ROS and NOX activity activate redox-sensitive pathways that favour RUNX2 expression, loss of the contractile phenotype, apoptosis and matrix-vesicle formation, thereby promoting osteogenic calcification [5,9,10,11,35].

### 6.8. ROS-Driven Signalling: NF-κB, MAPK, ER Stress and Calcification

ROS function as second messengers when tightly controlled, but persistent oxidative stress can sustain NF-κB and MAPK signalling and activate unresolved endoplasmic reticulum stress. These pathways promote inflammatory and osteoclastogenic transcription, impair osteoblast survival and, in vascular smooth-muscle cells, favour apoptosis and osteogenic reprogramming. Apoptotic bodies and matrix vesicles can then serve as sites of calcium–phosphate deposition, while RUNX2-related programmes reinforce the calcifying phenotype [6,9,10,48,51,58,59]. Table 3 summarises the principal experimental models, exposures and limitations.

### 6.9. Melatonin-Mediated Mitochondrial Protection and Redox Balance

Melatonin can accumulate within mitochondria, support electron-transport function and endogenous antioxidant defence, and limit excessive mitochondrial fission. In bone, MT2-linked signalling can restrain NF-κB and RANKL pathways, while vascular smooth-muscle-cell models show reduced calcification through AMPK/Drp1-dependent inhibition of mitochondrial fission [11,22,23,33,35]. These findings support a mechanistic role for melatonin in circadian–redox protection, but many experimental systems use pharmacological concentrations that are substantially higher than physiological nocturnal levels [35]. The principal melatonin actions and translational caveats are summarised in Table 4.

The therapeutic target, however, should be redox balance rather than maximal antioxidant capacity. Both oxidative distress and reductive stress can disrupt protein folding, mitochondrial adaptation and physiological signalling. Broad antioxidants may fail because they do not reach the relevant compartment, are given at the wrong disease stage, or suppress ROS signals required for host defence, exercise adaptation, autophagy and receptor function. A redox-aware strategy should therefore prioritise source-specific or compartment-targeted interventions, restoration of endogenous NRF2-responsive defence and chronobiologically appropriate timing, with biomarkers demonstrating target engagement [56]. This principle is depicted in Figure 5.

## 7. PTH Signalling in Cardiovascular Cells

PTH1R signalling has recognised cardiovascular actions, and hyperparathyroid states are associated with left-ventricular hypertrophy, vascular dysfunction and calcific disease [5,7,64]. Cardiovascular responses to PTH are complex and can show rapid desensitisation, so acute receptor physiology cannot be extrapolated directly to the sustained elevation of SHPT [5,65]. Most mechanistic studies also use defined PTH concentrations that are higher than typical circulating levels; consequently, the independent quantitative contribution of PTH to uraemic cardiomyopathy remains difficult to establish in patients [5].

### 7.1. Cardiomyocytes and Endothelium

In cardiomyocytes, PTH1R signalling can modify calcium handling and activate PKC-dependent hypertrophic pathways; clinical studies have reported associations between PTH and left-ventricular mass, although causality is not established [7,63,65]. In CKD, pressure and volume overload, RAAS activation and FGF23–FGFR4–calcineurin–NFAT signalling provide competing or interacting drivers of hypertrophy [1,5,7]. Endothelial and valvular endothelial models also indicate that PTH can activate ERK, p38 MAPK and NF-κB pathways and promote an osteogenic secretory phenotype [9]. These effects coexist with phosphate-related and broader CKD–MBD disturbances, which limits attribution of cardiovascular pathology to PTH alone [5,64,66].

### 7.2. Vascular Smooth-Muscle Cells and ER Stress

Vascular smooth-muscle cells are central effectors of medial calcification in CKD [63,64]. Under uraemic conditions, they can undergo osteochondrogenic transdifferentiation, increase RUNX2 and bone-matrix proteins, and release apoptotic bodies that facilitate mineral nucleation [64]. In cultured human aortic smooth-muscle cells, PTH induces apoptosis and activates PERK–CHOP and IRE1–JNK endoplasmic reticulum stress pathways; inhibition of JNK or CHOP attenuates the apoptotic response. The study supports ER stress as a possible mechanism linking PTH exposure to vascular calcification but does not establish direct PTH-driven mineralisation in patients [10]. PTH should therefore be viewed as a context-dependent contributor within a phosphate- and uraemia-dominated vascular environment, consistent with the absence of clear cardiovascular benefit in trials based solely on biochemical PTH lowering [5,10,63,64].

### 7.3. Circadian, Melatonin and Redox Overlays

Cardiovascular tissues are subject to circadian, autonomic and haemodynamic rhythms as well as nocturnal melatonin signalling. Melatonin can influence vascular tone and has antioxidant, anti-inflammatory and mitochondrial actions; in experimental vascular smooth-muscle cells, it attenuates calcification through AMPK/Drp1-dependent control of mitochondrial fission [11,22,23,34,35,67]. Whether loss of this temporal signal increases cardiovascular susceptibility to PTH, phosphate or uraemic oxidants in CKD has not been demonstrated clinically [5,35].

## 8. Integrated Mechanistic Model and Evidence Hierarchy

The links within the proposed axis differ markedly in evidential strength, as summarised in Table 3 and Table 5. Controlled human studies establish circadian and pul-satile organisation of PTH secretion, whereas the molecular parathyroid clock and its disruption in CKD–MBD have been demonstrated experimentally [16,17,19]. Human CKD and dialysis studies also support melatonin disruption, inflammation and oxida-tive stress, while impaired NRF2 defence is supported by experimental and transla-tional evidence [12,57,61]. Cell and animal studies establish that sustained PTH fa-vours RANKL-mediated resorption, whereas intermittent exposure can be anabolic [8,42,47], and that mitochondrial ROS and redox-sensitive pathways can drive in-flammatory, apoptotic and osteogenic responses [9,10,11]. These mechanistic studies frequently use supraphysiological exposures and therefore demonstrate biological ca-pability rather than the magnitude of pathway activity in human CKD [15,16,25].

The weakest links are the most integrative ones. CKD-associated immune dysfunction is well recognised, but the independent contribution of sustained PTH to specific innate or adaptive defects remains uncertain because available experimental, ex-vivo and observational studies cannot adequately separate PTH from the uraemic milieu [6,13,14]. Similarly, the hypothesis that circadian misalignment and melatonin loss amplify SHPT through redox effects in parathyroid, skeletal and cardiovascular tissues remains unproven in patients. No trial has shown that timed SHPT therapy, melatonin supplementation or targeted redox treatment improves fractures, vascular calcification, cardiovascular events or survival in CKD. The integrated circadian–melatonin–redox–immune–PTH model should therefore be interpreted as a graded, testable framework rather than an established causal pathway [6,14].

## 9. Clinical Implications and Future Research Directions

The clinical implications of this framework are prospective rather than prescriptive. Chronotherapy and redox-directed treatment remain research questions because the integrated axis has not yet been validated in human CKD–SHPT [16,56].

### 9.1. PTH Lowering and Hard Outcomes: A Disciplinary Anchor

EVOLVE provides an important clinical boundary for mechanistic interpretation. The trial randomised 3883 haemodialysis patients with moderate-to-severe SHPT to cinacalcet or placebo and, in the unadjusted intention-to-treat analysis, did not show a significant reduction in the primary composite of death or major non-fatal cardiovascular events [26]. Prespecified adjusted and lag-censored analyses suggested possible benefit, but they do not replace the neutral primary analysis [26]. Accordingly, improvement in mineral biomarkers or mechanistic pathways should not be assumed to translate into cardiovascular benefit without adequately powered outcome trials.

### 9.2. Chronotherapy of SHPT

Because PTH secretion and bone turnover are rhythmic, treatment timing is a reasonable experimental variable. Potential studies could compare timed and conventional dosing of active vitamin D analogues or calcimimetics and examine 24-h PTH profiles, bone-turnover markers, pharmacokinetics and safety. At present, however, chronotherapy for SHPT is supported by physiological rationale rather than clinical outcome evidence and should not guide routine prescribing [16,19].

### 9.3. Melatonin and Redox-Targeted Adjuncts

Melatonin improves sleep-related outcomes in some dialysis studies and is attractive for mechanistic trials because its timing, mitochondrial actions and receptor-mediated effects can be tested simultaneously [11,21,22,23,35]. It should not currently be presented as a treatment for SHPT, renal osteodystrophy or vascular calcification. Similarly, non-selective antioxidant supplementation cannot be recommended from elevated oxidative biomarkers alone. Future interventions should specify the ROS source or defence pathway, demonstrate target engagement, preserve physiological redox signalling and assess interactions with dialysis timing, iron administration, phosphate control and existing SHPT therapy [12,25,35,55]. A balanced appraisal must weigh the potential benefits against the drawbacks. A recent GRADE-assessed systematic review and meta-analysis of randomised controlled trials in CKD reported that melatonin supplementation modestly improved subjective sleep quality and reduced the lipid-peroxidation marker malondialdehyde, with a small increase in HDL-cholesterol. However, it had no significant effect on C-reactive protein, and the certainty of evidence was moderate for most outcomes [68]. Against these signals of benefit, safety considerations counsel caution: a large Japanese population-based cohort study examined hip-fracture risk associated with newer hypnotics, including the melatonin-receptor agonist ramelteon, underscoring that agents acting on melatonin pathways require explicit evaluation of skeletal and other safety outcomes in older and comorbid populations [69]. Further drawbacks specific to advanced CKD include altered melatonin pharmacokinetics with impaired clearance and possible accumulation, uncertainty over optimal formulation, dose and circadian timing, and the absence of trials powered for fracture, cardiovascular or mortality endpoints. On balance, current evidence supports melatonin as a candidate for well-designed mechanistic and outcome trials in CKD–SHPT rather than as an established therapy.

### 9.4. Circadian- and Redox-Aware Biomarkers

Future studies should move beyond isolated random PTH measurements and characterise temporal and biochemical phenotypes. Candidate measures include 24-h PTH profiles, time-standardised bone-turnover markers, ambulatory blood-pressure dipping, actigraphy or dim-light melatonin onset, and imaging of vascular calcification or left-ventricular mass. Redox phenotyping could incorporate validated markers such as F2-isoprostanes, glutathione redox status or NRF2-responsive signatures, sampled at standardised times relative to sleep, meals and dialysis. Exploratory immune phenotyping may include leukocyte subsets and functional assays, but these signals cannot be interpreted as PTH-specific without adjustment for infection, dialysis modality, vitamin D status and uraemic burden [6,13]. Such multimodal panels require prospective validation before they can guide treatment [16,32,52,63].

## 10. Knowledge Gaps and Future Directions

### 10.1. Novel Contribution and Falsifiable Predictions

This review advances four linked propositions that have not previously been organised as a single testable CKD–SHPT model. First, it treats secretory timing—not only mean PTH concentration—as a candidate disease variable and predicts CKD-stage- and dialysis-schedule-specific uncoupling among the parathyroid clock, the nocturnal melatonin signal and bone-turnover rhythms. Second, it identifies the parathyroid chief cell as the missing redox compartment and predicts that mitochondrial or NOX-derived ROS, together with inadequate NRF2 adaptation, contributes to loss of CaSR/VDR/FGFR1–Klotho sensing before or during nodular selection. Third, it separates upstream uraemic inflammation from the less certain reciprocal PTH–immune arm and requires cell-specific, concentration-resolved evidence before causality is assigned. Fourth, it makes timing, ROS source, cellular compartment and patient-relevant exposure explicit design variables for intervention studies. The framework is novel because these predictions can be falsified independently; failure of one link does not validate or invalidate the others [6,25]. Crucially, this framework is distinguished from prior reviews that have addressed related themes in isolation. Earlier syntheses have characterised inflammation and oxidative stress largely as downstream consequences of uraemia and dialysis [12], surveyed NRF2–KEAP1 biology in kidney disease without connecting it to parathyroid function [27,61], examined immune mechanisms in CKD–MBD without a temporal dimension [6], or considered melatonin principally as a chronobiotic and sleep aid in CKD [21,54]. This review is novel in three specific respects: it identifies the parathyroid chief cell as a discrete and directly testable redox compartment; it couples the parathyroid molecular clock to loss of the nocturnal melatonin signal as an explicit, falsifiable prediction rather than a general association; and it frames the reciprocal PTH–immune arm as deliberately hypothesis-generating, specifying the concentration-resolved, cell-specific evidence required before causality can be assigned [6,17].

### 10.2. Experimental and Clinical Priorities

The first priority is descriptive: 24-h PTH profiles, melatonin timing and, where feasible, parathyroid clock biology should be characterised across CKD stages and dialysis schedules [17]. Mechanistic studies should then determine whether mitochondrial ROS, specific NOX isoforms, NRF2 failure or ER stress contribute causally to receptor loss and parathyroid hyperplasia, while peripheral models should separate PTH1R effects from phosphate, FGF23, cytokines and uraemic toxins at clinically relevant exposures. The immune arm requires the same approach, with monocyte/macrophage, neutrophil and lymphocyte studies controlling for calcium, phosphate, vitamin D, uraemic toxins and dialysis exposure [6,13]. Interventional studies could subsequently compare timed and conventional SHPT therapy, test melatonin with prespecified formulation and circadian timing, and evaluate source-specific redox interventions with target-engagement biomarkers. Any such strategy must account for dialysis-related oxidative perturbations and ultimately demonstrate effects on clinically meaningful skeletal, cardiovascular or survival outcomes before adoption in practice [16,56]. Table 6 summarises these investigational directions.

## 11. Limitations

This is a mechanism-focused narrative review rather than a systematic review, so study selection and interpretation are susceptible to selection bias and no formal risk-of-bias assessment or quantitative synthesis was performed. The evidence base is heterogeneous, combining human physiological and clinical observations with cell and animal studies that often use PTH, phosphate, melatonin or oxidant exposures above those encountered clinically. Direct evidence linking oxidative stress to human parathyroid chief-cell dysfunction is particularly limited. Reported melatonin profiles in advanced CKD are also inconsistent, with attenuation, phase disturbance and possible accumulation described in different settings. Finally, the neutral unadjusted primary outcome of EVOLVE limits any inference that biochemical PTH lowering alone improves hard cardiovascular outcomes [6,26].

## 12. Conclusions

SHPT in CKD is primarily a disorder of mineral feedback, but its expression occurs within a temporally organised and redox-sensitive biological system. The framework developed here links the experimental parathyroid clock, disturbed nocturnal melatonin signalling, compartment-specific ROS/NRF2 imbalance and a cautiously defined reciprocal PTH–immune component. Uraemic toxins, hyperphosphataemia, inflammation and dialysis-related exposures provide established sources of oxidative stress, whereas direct redox-driven chief-cell dysfunction and independent immune effects of PTH remain unproven in humans. The immediate research priorities are therefore to obtain time-resolved PTH and melatonin profiles, define chief-cell redox biology directly, use patient-relevant experimental exposures and test whether treatment timing changes pharmacodynamic or clinical responses. Chronotherapy, melatonin supplementation and targeted redox or immune interventions should remain investigational until CKD trials demonstrate clinically meaningful benefits.

## Figures and Tables

**Figure 1:**
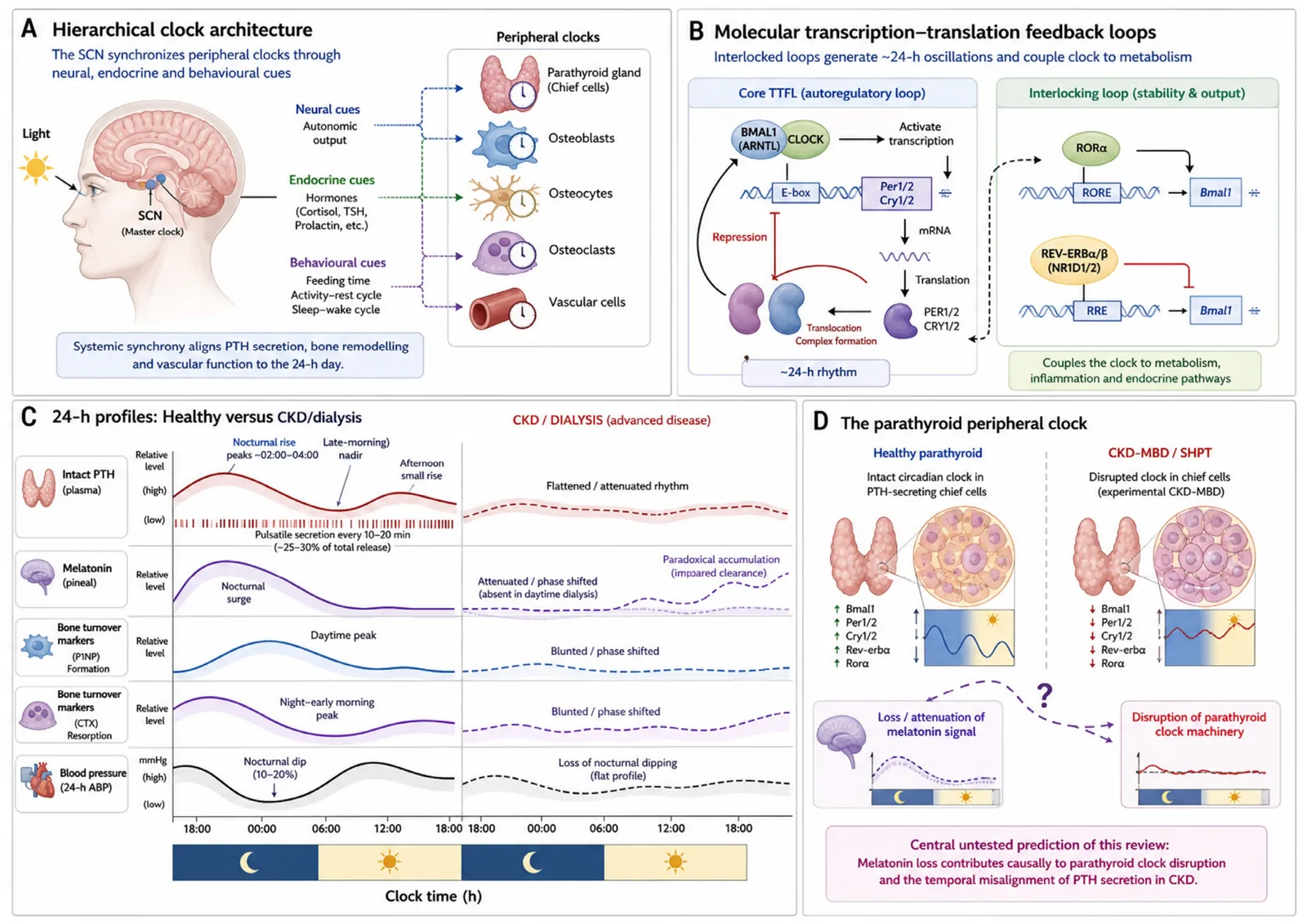
Circadian organisation of PTH secretion and melatonin signalling in health and in advanced CKD. Four-panel figure illustrating the temporal framework of the proposed axis. (**A**) Hierarchical circadian organisation: the hypothalamic suprachiasmatic nucleus (SCN) synchronises peripheral clocks in the parathyroid gland, bone and vascular tissues through neural, endocrine and behavioural cues. (**B**) Molecular clock: BMAL1/CLOCK drives PER and CRY transcription, with PER/CRY feedback inhibition and REV–ERB/ROR regulation stabilising the cycle. (**C**) Healthy temporal pattern: PTH rises during the biological night while melatonin peaks nocturnally. (**D**) Advanced CKD: sleep–light disruption, dialysis scheduling and altered peripheral-clock function are associated with disturbed melatonin and PTH timing. The direct coupling between melatonin loss and the parathyroid clock in human CKD remains hypothetical. For consistency, panels C and D use the same schematic biphasic PTH pattern, with a dominant nocturnal/early-morning peak and a smaller afternoon rise. (Solid arrows indicate confirmed relationships, whereas dashed arrows indicate possible or proposed relationships).

**Figure 2:**
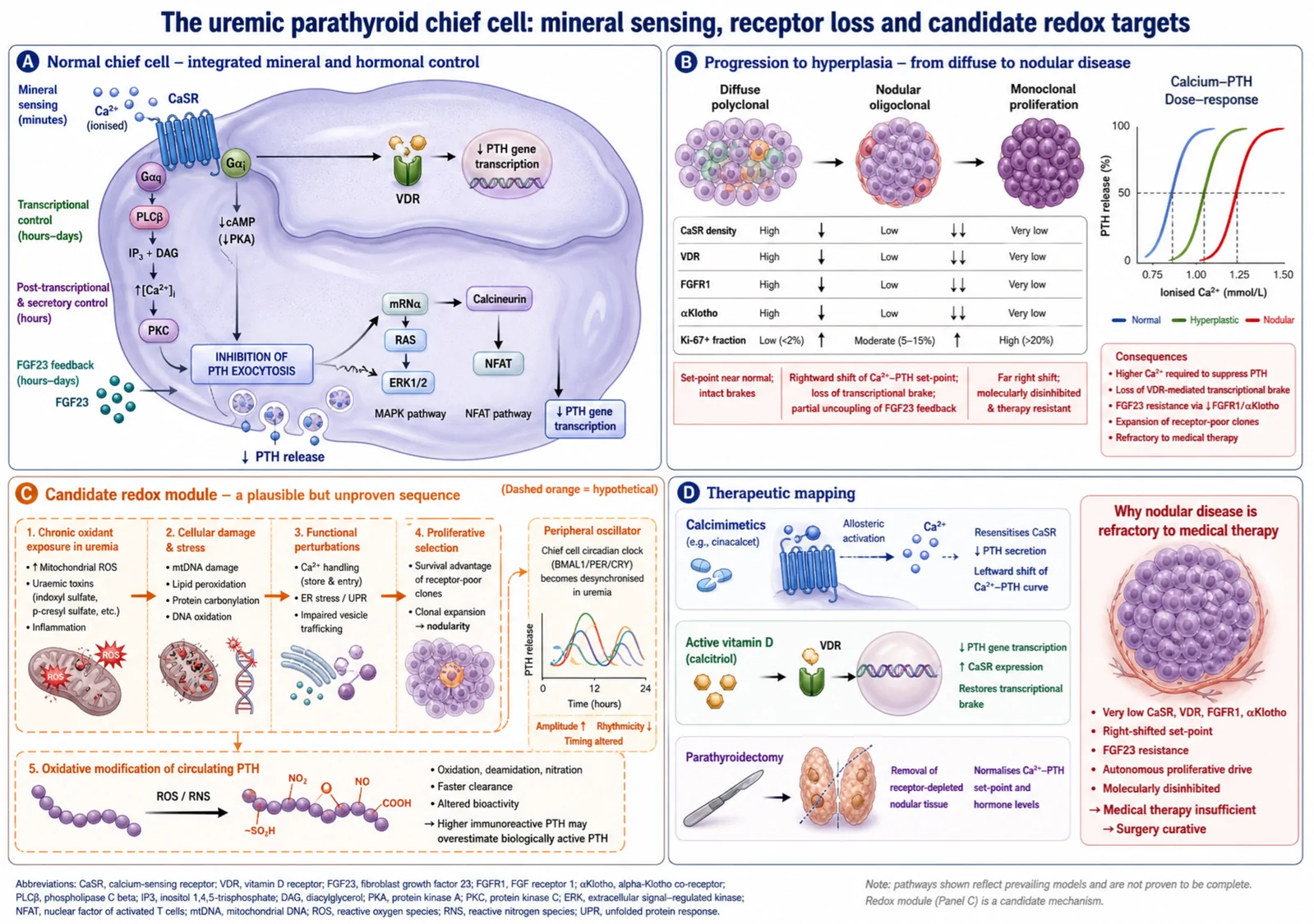
The uraemic parathyroid chief cell: mineral sensing, receptor loss and candidate redox targets. (**A**) Normal chief cell. Extracellular ionised calcium is sensed by CaSR, while calcitriol–VDR and FGF23–FGFR1-Klotho pathways suppress PTH synthesis and secretion. (**B**) CKD–SHPT. Persistent mineral stress promotes hyperplasia and reduced CaSR, VDR, FGFR1 and Klotho expression, weakening inhibitory feedback. (**C**) Candidate redox mechanisms. Uraemic oxidants, phosphate and inflammatory mediators may increase mitochondrial and NOX-derived ROS and alter NRF2-responsive defence. These redox effects are proposed targets for study rather than established causes of human parathyroid hyperplasia. (**D**) Research read-outs include time-resolved PTH secretion, receptor abundance, mitochondrial function, NOX activity, NRF2 target genes and markers of ER stress.

**Figure 3:**
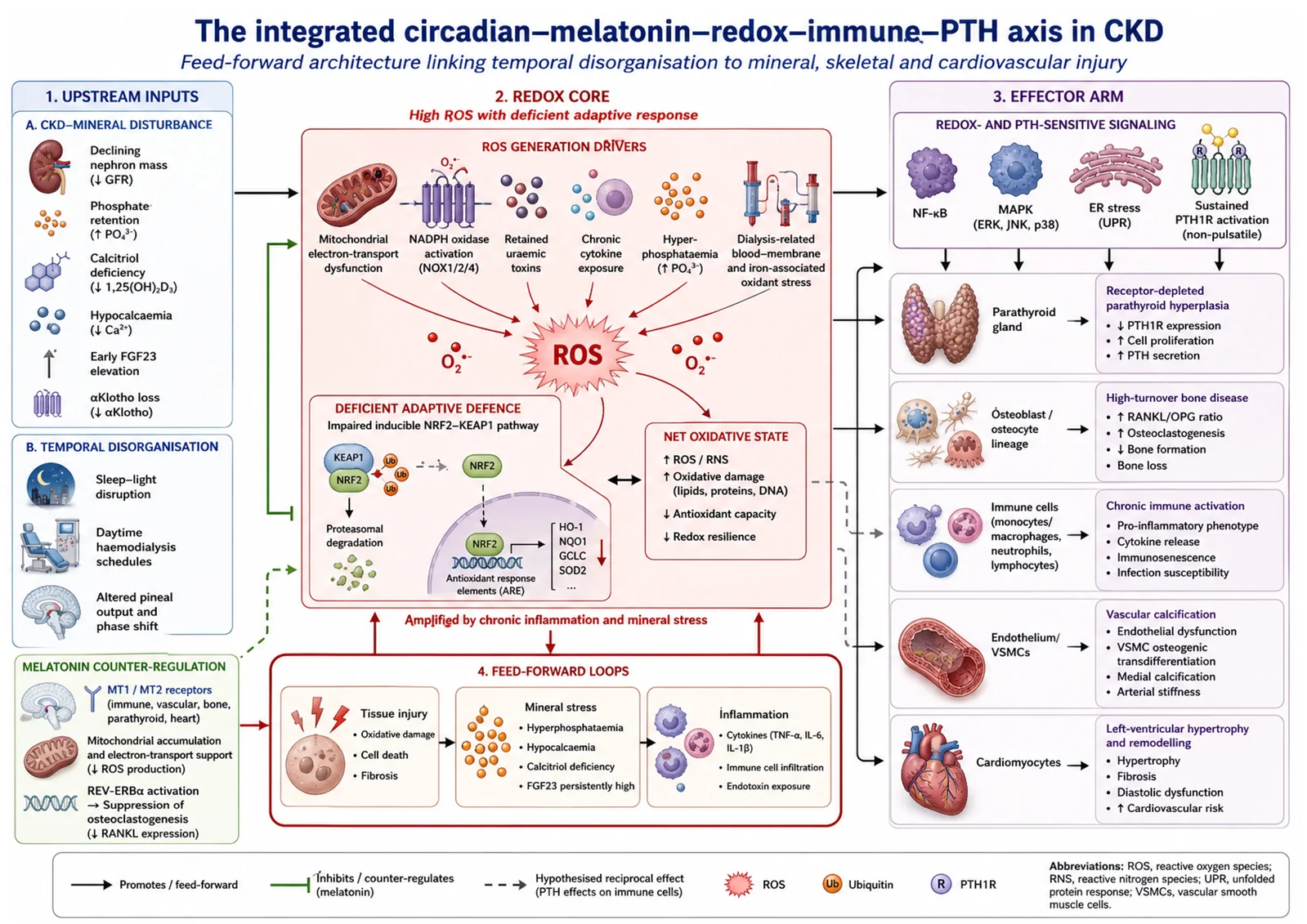
The integrated circadian–melatonin–redox–immune–PTH axis in CKD. Schematic of the proposed feed-forward model. Declining kidney function produces phosphate retention, calcitriol deficiency, rising FGF23 and progressive SHPT. Circadian disruption and altered nocturnal melatonin signalling may weaken temporal and mitochondrial protection, while uraemic toxins, inflammation, hyperphosphataemia and dialysis-related exposures increase oxidative stress. Redox-sensitive NF-κB, MAPK and ERstress pathways can then contribute to skeletal, vascular and inflammatory injury. A reciprocal effect of sustained PTH on immune-cell function is shown as a hypothesis-generating link because direct evidence in CKD–SHPT remains limited. Solid arrows should denote established or well-supported relationships; dashed arrows should denote proposed or incompletely validated interactions. *Abbreviations:* CKD, chronic kidney disease; ER, endoplasmic reticulum; FGF23, fibroblast growth factor 23; LVH, left-ventricular hypertrophy; mtROS, mitochondrial reactive oxygen species; NOX, NADPH oxidase; OPG, osteoprotegerin; PTH, parathyroid hormone; RANKL, receptor activator of NF κB ligand; ROS, reactive oxygen species; SHPT, secondary hyperparathyroidism; VSMC, vascular smooth muscle cell.

**Figure 4:**
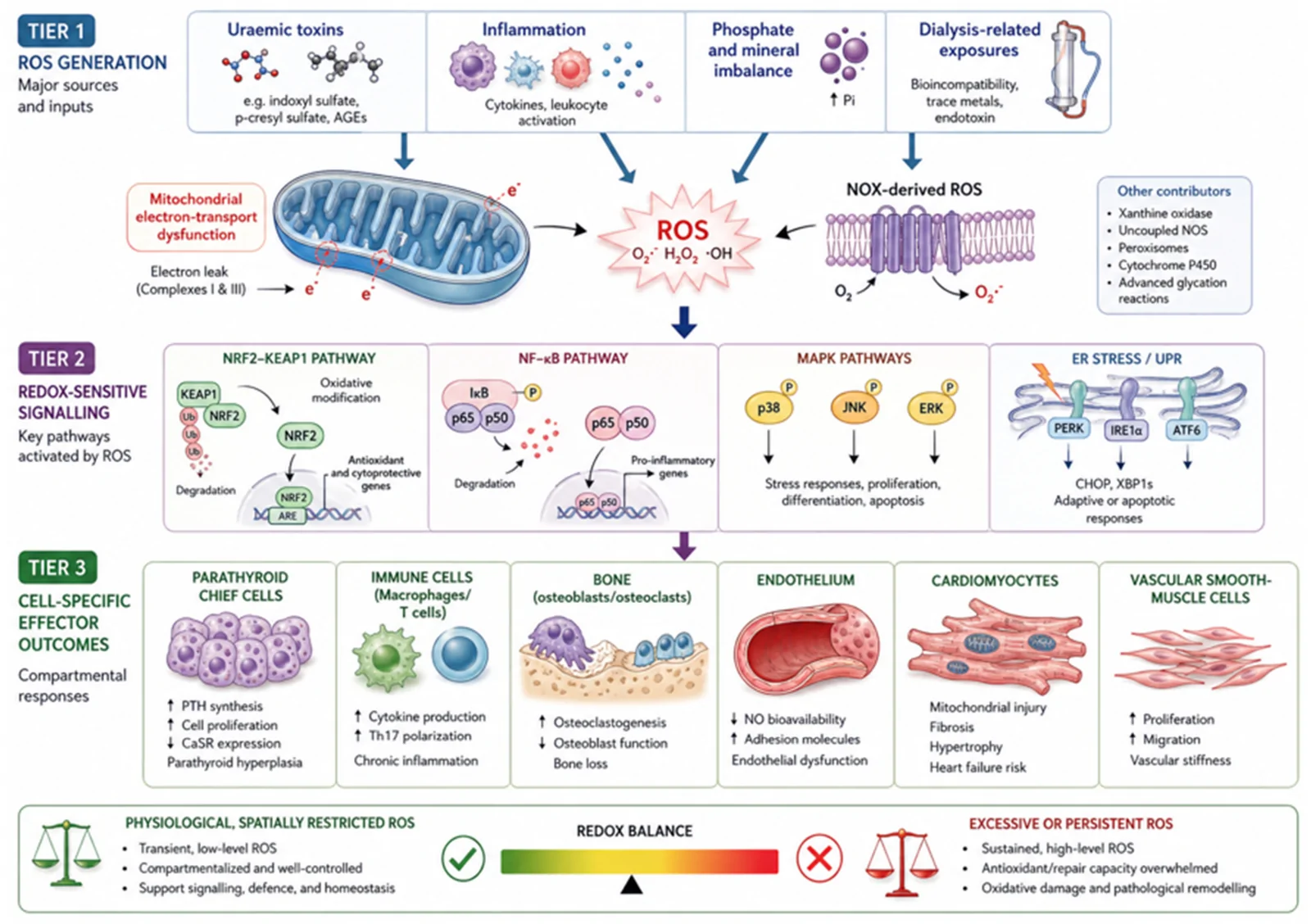
ROS sources, redox-sensitive transduction and cell-specific effector outcomes. Three-tier figure linking ROS generation to downstream tissue responses. Tier 1 shows mitochondrial electron-transport dysfunction and NOX-derived ROS, with uraemic toxins, inflammation, phosphate and dialysis-related exposures as major inputs. Tier 2 shows redox-sensitive signalling through NRF2–KEAP1, NF–κB, MAPK and ER stress pathways. Tier 3 shows compartment-specific outcomes in parathyroid chief cells, immune cells, bone, endothelium, cardiomyocytes and vascular smooth-muscle cells. Physiological, spatially restricted ROS should be distinguished from excessive or persistent ROS that overwhelm antioxidant and repair capacity.

**Figure 5:**
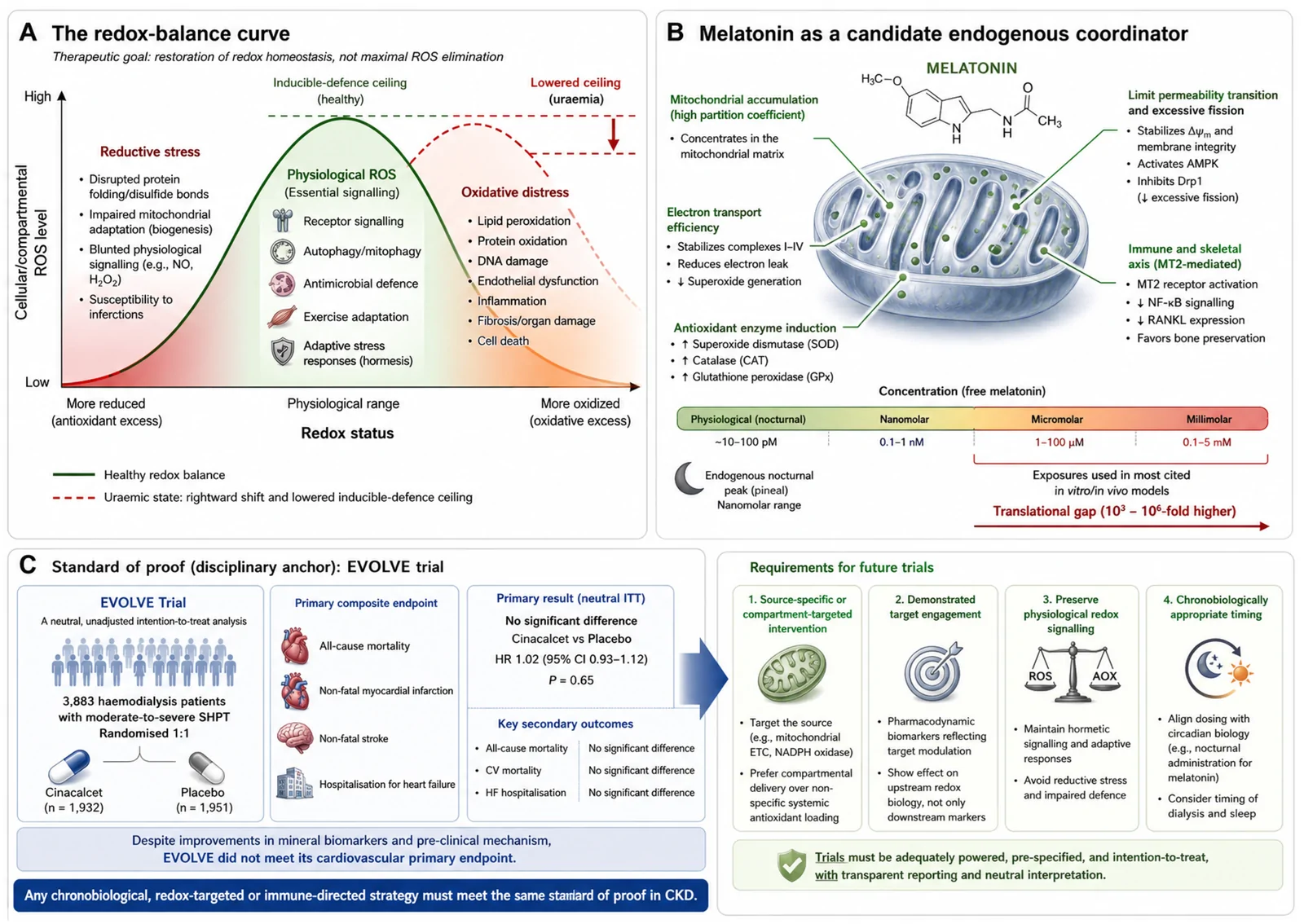
Redox balance, melatonin counter-regulation and the translational standard of proof. (**A**) Redox balance. Low, compartmentalised ROS support normal signalling and adaptive stress responses, whereas excessive oxidative or reductive pressure impairs cellular function. (**B**) Melatonin counter-regulation. Receptor-dependent and mitochondrial actions may support antioxidant defence, limit excessive mitochondrial fission and modulate inflammatory signalling. (**C**) Translational hierarchy. Mechanistic and biomarker findings should progress through target-engagement studies and adequately controlled CKD trials before therapeutic claims are made. The neutral unadjusted primary cardiovascular result of EVOLVE is shown as a reminder that biochemical improvement does not necessarily translate into hard clinical benefit.

**Table 1:** Integrated components of the circadian–melatonin–redox–immune–PTH axis.

Component	Principal Disturbance in CKD	Mechanistic Consequence	Evidence Base (Level; Species)
Circadian clock	Sleep–light disruption; altered peripheral-clock amplitude	Disordered timing of PTH, metabolism, inflammation and repair	Human physiology + experimental CKD [16,17,25,29,30,32]
Melatonin	Attenuated or phase-shifted nocturnal signal	Reduced clock reinforcement, mitochondrial support and antioxidant-enzyme activation	Human sleep studies + preclinical mechanisms [22,23,25,33,35,54]
Redox system	mtROS and NOX activation; impaired NRF2–KEAP1 response	NF-κB/MAPK activation, ER stress, apoptosis and macromolecular damage	Experimental CKD + human biomarkers [12,27,55,56,57]
Parathyroid gland	CaSR/VDR/FGFR1–Klotho loss; hyperplasia; disturbed clock	Sustained PTH secretion and reduced inhibitory feedback	Human tissue + animal models [5,17,36,37,38]
Immune system	Chronic innate/adaptive activation; uraemic and dialysis-related leukocyte stress	Cytokine and ROS amplification, impaired host defence and possible reciprocal modulation by sustained PTH	Human CKD immune dysfunction + heterogeneous PTH studies [6,13,14]
Bone	Continuous PTH plus oxidative injury	RANKL induction, osteoclastogenesis, osteoblast/osteocyte dysfunction	Strong cell/animal; indirect clinical [8,42,43,44,47,51]
Cardiovascular tissues	PTH, phosphate and uraemic oxidants	LVH, endothelial dysfunction, VSMC apoptosis and calcification	Cell/animal + clinical association [5,7,9,10,11,58,59,60]

**Table 2:** Cell-specific oxidative and immune effects within the proposed axis.

Cell Type	Redox Insult (Typical Model)	Key Pathways	Expected Consequence	Evidence Tier; Limitation
Parathyroid chief cell	Uraemic oxidants, phosphate, mitochondrial stress	NRF2 failure; ER stress; DNA/protein oxidation	Impaired sensing, proliferative selection, PTH dysregulation	Hypothesised; direct human data lacking [17,27,36,61]
Monocyte/macrophage	Uraemic toxins, cytokines, ROS and sustained PTH exposure	Ca^2+^ signalling; NF-κB; inflammasome; ROS	Altered activation, polarisation and cytokine output	Experimental/ex-vivo; direct PTH contribution uncertain [6,13,14]
Neutrophil	Uraemia, dialysis and PTH-associated intracellular Ca^2+^ disturbance	Chemotaxis; phagocytosis; oxidative burst	Impaired microbial killing and host defence	Older ex-vivo evidence; strong uraemic and dialysis confounding [6,13]
T- and B-lymphocyte	Uraemia, vitamin D deficiency and PTH exposure	PTH-responsive cAMP/Ca^2+^ signalling; activation and proliferation	Altered cellular and humoral immune responses	Inconsistent experimental/ex-vivo findings; CKD–SHPT causality unproven [6,13]
Osteoblast	mtROS, cytokines, sustained PTH (cultures)	FOXO/Wnt competition; JNK/p38; apoptosis	Reduced differentiation and matrix formation	Cell/animal; supraphysiological exposures [14,44,48,51]
Osteocyte	Mitochondrial dysfunction, oxidative DNA damage	NF-κB/MAPK; apoptosis; RANKL induction	Remodelling signals and increased resorption	Cell/animal; limited in-vivo CKD data [47,51]
Endothelial cell	NOX/mtROS; uraemic toxins	eNOS uncoupling; NF-κB; apoptosis	Reduced nitric oxide, inflammation, dysfunction	Cell/animal + human biomarkers [9,12,56]
Cardiomyocyte	mtROS plus Ca^2+^/PKC stress	MAPK; calcineurin–NFAT; ER stress	Hypertrophy, energetic failure and apoptosis	Cell/animal; PTH contribution hard to isolate [5,7,63]
VSMC	High phosphate, mtROS and NOX	NF-κB/AKT/RUNX2; PERK–CHOP; IRE1–JNK	Osteogenic switch, apoptosis and calcification	Reproducible cell/animal evidence; phosphate is a major driver [6,9,10,48,51,58,59]

**Table 3:** Model, exposure and evidence strength for principal mechanisms. Concentrations are representative of the cited experimental models and are frequently supraphysiological relative to circulating human values. (Arrowheads indicate the temporal sequence of biological events.).

Mechanism/Claim	Model (Species)	Exposure (Concentration/Duration)	CKD Relevance	Evidence Strength	Principal Limitation
Continuous vs. intermittent PTH sets RANKL/resorption balance	Rodent bone; osteoblast/osteocyte cultures	PTH(1–34) ~10^−8^–10^−7^ M; continuous vs. pulsed	Models the sustained exposure of SHPT	Established (animal/cell)	Human SHPT exposure is lower and more variable [8,42,43]
PTH -> VSMC apoptosis via ER stress; possible link to calcification	Human aortic SMC	PTH ~10^−7^ M ± 2–3 mmol/L Pi	Direct human-cell relevance	Mechanistically supported (human cells)	In-vitro PTH exposure; direct mineralisation and clinical causality not established [10]
High phosphate → mtROS/NF-κB → calcification	Human/rodent VSMC; CKD rodents	Pi 2–3 mmol/L or β-glycerophosphate ~10 mM	High (uraemic hyperphosphataemia)	Established (cell/animal)	Experimental phosphate exposure may exceed usual serum concentrations [58,59]
Oxidative stress → RUNX2/AKT → calcification	VSMC	H_2_O_2_ ~100–200 µM (bolus)	Indirect (uraemic-oxidant surrogate)	Mechanistically supported (cell)	Bolus oxidant exposure differs from endogenous compartmentalised ROS [58,59]
Impaired NRF2–KEAP1 inducible defence	CKD rodents; human biomarkers	Chronic renal failure models	High	Established (animal) + human biomarkers	Evidence is strongest experimentally; effects are tissue- and context-dependent [61]
Melatonin → mitochondrial protection/calcification	VSMC; rodent vascular models	Melatonin ~µM–mM	Hypothetical in CKD	Preclinical	Preclinical; dose and CKD translation remain uncertain [11,22,23,33,35]
Melatonin/parathyroid–clock coupling	Experimental CKD–MBD (rodent)	Genetic/disease models	Hypothesised in humans	Preclinical/hypothesis	Not directly demonstrated in human CKD; coupling remains a hypothesis [16,17,18,19,20,21,22]
PTH → immune-cell activation and effector function	Immune-cell cultures; ex-vivo patient cells	PTH or fragments, often µM; mixed uraemic milieu	Hypothesis-generating	Experimental/ex-vivo	Heterogeneous PTH fragments/concentrations and uraemic confounding [6,13,14]

**Table 4:** Melatonin actions relevant to redox and tissue protection.

Target	Proposed Action	Mechanism	Model and Concentration	Evidence; Limitation
Mitochondria	Preserves membrane potential and electron transport; limits fission	Direct mitochondrial accumulation; AMPK/Drp1 quality control	VSMC/vascular models; µM–mM	Preclinical [11,22]; supraphysiological dose
Antioxidant defence	Increases SOD, catalase, glutathione peroxidase and glutathione pathways	Transcriptional/enzyme regulation; indirect antioxidant action	Cell/animal; µM–mM	Preclinical/translational [22]; CKD data limited [35]
Bone	Supports osteoblastogenesis; reduces RANKL-dependent osteoclastogenesis	MT2, MEK1/2, MEK5, REV-ERBα, NF-κB	Cocultures + small non-CKD RCTs; µM in vitro	Preclinical + non-CKD RCTs [23,24,33,35,50,51]
Vasculature	Reduces inflammation and mitochondrial dysfunction	MT receptors; AMPK and redox pathways	Rodent/VSMC models; µM–mM	Predominantly preclinical [11,14,22,34,35]
Circadian system	Reinforces biological-night signalling	SCN/peripheral-clock entrainment	Human chronobiology; physiological–pharmacological	Established chronobiology; CKD effects uncertain [25,29,32,54]

**Table 5:** Evidence strength for major claims.

Claim	Evidence Grade	Basis (Species)	Interpretive Limit
PTH secretion is circadian and partly governed by a parathyroid clock	High	Controlled human PTH physiology + experimental parathyroid–clock studies [16,17,19]	CKD-stage-specific human profiles remain sparse
Melatonin rhythm is disturbed in advanced CKD	Moderate	Human observational/interventional CKD sleep studies [21,52,53,54]	Direction and clearance effects vary by study
CKD/dialysis cause oxidative stress and impaired NRF2 defence	Moderate–high	Human biomarkers + experimental causality [12,27,56,57,61]	Biomarker heterogeneity; compartment specificity
ROS drive RANKL, ER stress and vascular calcification	High (mechanistic)	Cell/animal experiments [6,9,10,48,51,58,59]	Cell-specific effects are inconsistent; no intervention demonstrates independent reversibility.
Sustained PTH independently disrupts immunity in CKD–SHPT	Low	Experimental/ex-vivo and observational literature [6,13,14]	Direction and independence from uraemia, phosphate and dialysis remain uncertain
Melatonin protects mitochondria and activates antioxidant enzymes	Moderate (mechanistic)	Broad preclinical literature; limited human outcomes [11,22,23,33,35]	Dose, timing and CKD translation unresolved
Integrated axis improves hard outcomes when targeted	Very low	No dedicated outcome trial [26]	Hypothesis-generating only

**Table 6:** Investigational chronotherapeutic and redox strategies (research directions, not current clinical recommendations).

Strategy	Rationale	Required Trial Features	Current Status
Timed calcimimetic or active vitamin D	Align PTH suppression with secretory rhythm; avoid oversuppression	24-h PTH/turnover profiles; PK–PD; safety	Investigational [16,18,26,32]
Timed melatonin	Restore nocturnal signal; test mitochondrial/redox engagement	Circadian phenotype; formulation/dose; sleep + redox + mineral endpoints	Sleep evidence only; SHPT benefit unproven [22,35]
NOX- or mitochondria-targeted therapy	Reduce pathological ROS at source while preserving signalling	Cell/source biomarkers; dialysis context; clinical outcomes	Preclinical/early translational [11,35,55,56]
NRF2-pathway restoration	Rebuild endogenous adaptive defence	Dose-response and safety; avoid chronic overactivation	Investigational [12,27,56]
Dialysis optimisation	Reduce membrane-, iron- and treatment-cycle oxidative stress	Modality/membrane randomisation; timed sampling	Context-dependent [14,52,53]
Integrated stratification	Identify a circadian–redox-high phenotype	PTH rhythm, melatonin, dipping, redox markers, imaging	Investigational [18,19,26,32,60]

## Data Availability

No new data were created or analysed in this study.
